# Patterns and Frequency of Pathogenic Germline Mutations among Patients with Newly-Diagnosed Endometrial Cancer: The Jordanian Exploratory Cancer Genetics (Jo-ECAG) Endometrial Study

**DOI:** 10.3390/cancers16142543

**Published:** 2024-07-15

**Authors:** Hikmat Abdel-Razeq, Hira Bani Hani, Baha Sharaf, Faris Tamimi, Hanan Khalil, Areej Abu Sheikha, Mais Alkyam, Sarah Abdel-Razeq, Tala Ghatasheh, Tala Radaideh, Suhaib Khater

**Affiliations:** 1 Department of Internal Medicine, King Hussein Cancer Center, Amman 11941, Jordan; hb.14507@khcc.jo (H.B.H.); bs.13628@khcc.jo (B.S.); ft.10481@khcc.jo (F.T.); hk.10660@khcc.jo (H.K.); asheikha@khcc.jo (A.A.S.); ma.15435@khcc.jo (M.A.); talamghatasheh@gmail.com (T.G.); talaradaideh97@gmail.com (T.R.); sk.13840@khcc.jo (S.K.); 2 School of Medicine, The University of Jordan, Amman 11942, Jordan; sarahrazeq@gmail.com

**Keywords:** endometrial cancer, pathogenic germline variants, variants of uncertain significance, VUS, inherited cancer

## Abstract

**Simple Summary:**

Endometrial cancer is a common cancer among women worldwide. In rare occasions, it can be caused by mutations in genes that can be inherited, and, mostly, as part of the Lynch syndrome that can also increase the risk of developing other cancers, mostly colorectal. In this study, we analyzed genetic alterations in 130 Arab patients with endometrial cancer. Among the whole group, 18 (13.8%) patients had positive mutations in *MLH1*, *PMS2*, *MSH2*, *ATM*, *MUTYH*, and *BRCA2*. Such mutations were more common in younger patients and in patients with specific endometrial cancers like carcinosarcoma and clear cell carcinoma.

**Abstract:**

Most of endometrial cancers are sporadic, with 5% or less being attributed to inherited pathogenic germline mutations and mostly related to the Lynch syndrome. To our knowledge, this is the first study to investigate patterns and frequencies of germline mutations in patients with endometrial cancer in an Arab region. Consecutive patients with endometrial cancer (n = 130), regardless of their age and family history, were enrolled. Germline genetic testing, using an 84-gene panel, was performed on all. Almost half of the patient population (n = 64, 49.2%) was tested based on international guidelines, while the remaining patients (n = 66, 50.8%) were tested as part of an ongoing universal germline genetic testing program. Among the whole group, 18 (13.8%) patients had positive pathogenic or likely pathogenic (P/LP) germline variants. The most common variants encountered were in *MLH1* (n = 4, 22.2%), *PMS2* (n = 3, 16.7%), *ATM*, *MSH2*, *MUTYH*, and *BRCA2* (n = 2, 11.1% each). In addition, three (2.3%) patients were found to have an increased risk allele of the *APC* gene. P/LP variants were more common among patients with carcinosarcoma and clear cell carcinoma, younger patients (age ≤ 50 years), and in patients with a non-metastatic disease. We conclude that germline genetic variants, mostly in genes related to the Lynch syndrome, are relatively common among Arab patients with endometrial cancer.

## 1. Introduction

Endometrial cancer is the second most common gynecologic malignancy, with over 420,000 cases diagnosed in 2021 [1,2]. Most cases are sporadic, with 5% or less being attributed to inherited pathogenic germline mutations which tend to occur in patients that are 10 to 20 years younger than patients affected by the sporadic ones [3].

Endometrial carcinoma is classified, based on its phenotype, into two groups. Type 1, referred to as endometrioid carcinoma, is the most common type and is usually related to a state of estrogen excess. The microscopic appearance of the tumor resembles that of the proliferative endometrium, with a variable degree of glandular complexity and cellular pleomorphism. Type 2 endometrial cancer includes all other histological subtypes, including grade 3 endometrioid carcinoma, clear cell carcinoma, papillary serous carcinoma, and undifferentiated carcinoma. Because of significant overlap in the histopathological features of endometrial tumors, molecular analyses and subtype classification may help select appropriate therapies [4,5]. Uterine serous carcinoma, which represents less than 10% of all endometrial cancers, is an aggressive subtype, frequently present with advanced-stage diseases and associated with a high recurrence rate among patients who present with earlier stages. The 5-year overall survival rate may not exceed 40% [6,7,8,9].

Clinicians tend to pay more attention to the histological type, disease stage, and treatment options of endometrial cancers and may pay less attention to issues related to family history and cancer-predisposing syndromes [10,11,12].

Screening of the tumor for defective DNA mismatch repair (MMR) using immunohistochemistry (IHC) and/or microsatellite instability (MSI) may be used to direct physicians towards the identification of patients who should undergo mutation testing for the Lynch syndrome [13,14,15]. The Cancer Genome Atlas (TCGA) study was conducted on 373 endometrial carcinomas with various subtypes, including low-grade and high-grade endometrioid and serous carcinomas. The study classified, based on somatic molecular alterations, four subtypes of endometrial cancers: *POLE* (DNA polymerase epsilon) ultramutated [16], microsatellite instability hypermutated (MSI-H), copy number low (wild type *P53*), and copy number high (abnormal *P53*) which is characterized by higher incidence of *TP53* alterations [17]. This genomic classification, which can be linked to a specific phenotype, is endorsed by many international guidelines including the National Comprehensive Cancer Network (NCCN) [18]. *POLE*-mutation-associated endometrial cancers are usually high-grade tumors with deep myometrial and lymphovascular invasion, and tend to have a good prognosis [16,19]. Endometrial cancers with the *p53* mutant constitute the most aggressive subtype and require a multimodality treatment, including chemotherapy. Tumors with MSI-H have an intermediate prognosis and can be associated with cancer-predisposing germline mutations [17]. 

The Lynch syndrome is the most common genetic alteration encountered in patients with endometrial cancer. The Lynch syndrome, also known as Hereditary Nonpolyposis Colorectal Cancer (HNPCC) syndrome, is an autosomal dominant disorder that can be transmitted through paternal or maternal lineages and is caused by inherited pathogenic variants in the highly penetrant mismatch repair genes *MLH1, MSH2, MSH6, PMS2,* and *EPCAM* [20]. In addition to colorectal cancer, individuals with the Lynch syndrome are at higher risk of developing cancers of the endometrium, ovary, breast, stomach, small bowel, biliary tract, pancreas, urinary tract, prostate, and skin [21]. The syndrome accounts for 2% to 5% of all colorectal cancers and increases the lifetime risk of colorectal cancer to as much as 60% to 85% [22]. Endometrial cancers occurring in association with the Lynch syndrome tend to occur at a younger age and are known for their association with specific molecular features including MMR deficiencies and presence of MSI. 

For patients with the Lynch syndrome, the lifetime risk of developing endometrial cancer is approximately 30–70% [20,21,22,23]. Other studies have reported that 60% of women with the Lynch syndrome present initially with gynecological cancers, mostly endometrial [24]. 

Little is known about non-Lynch pathogenic variants associated with endometrial cancer. To our knowledge, this is the first study to investigate patterns of endometrial cancer genetics in an Arab region.

## 2. Methods

This is a prospective study in which all consecutive patients with a confirmed diagnosis of endometrial cancer, regardless of their age and family history, were invited to participate. All patients were adults (≥18 years), and all were treated and followed up at King Hussein Cancer Center. Patients who had another primary cancer diagnosed prior to the endometrial cancer were excluded. Germline genetic testing using an 84-gene panel was performed using a peripheral blood sample and a next generation sequencing (NGS) platform; details related to the procedure have been described previously [25] (Table S1). 

Multigene panel testing, including full gene sequencing, duplication and deletion analysis, and variant interpretation, were performed at a single commercial reference laboratory (Invitae Corporation, San Francisco, CA, USA). If only one pathogenic germline variant was identified in an autosomal recessively inherited gene, the results were labeled as carrier and were not included in the positive rate. For our analysis, results were considered to be VUS when at least one VUS was identified in the absence of positive pathogenic or likely pathogenic variants in the same patient. 

All patients were evaluated by one of the investigators during their first clinical encounter at the specialty medical oncology clinic, following which the patients were referred to one of our genetic counselors. Study procedures were explained, and the consequences of genetic testing were thoroughly discussed with the patient herself and close relatives, when requested. Variants were interpreted based on guidelines from the American College of Medical Genetics and Genomics classification, and pathogenic (P), likely pathogenic (LP), and variants of uncertain significance (VUS) were reported to clinicians [26,27]. All GGT results were reviewed by genetic counselors and disclosed to the patient by their oncologist. Patients with pathogenic/likely pathogenic variants and at-risk family members were offered genetic counseling.

Clinical data, including demographics and medical and family history were collected using an electronic case report form based on a review of the institution’s electronic medical record. All data were deidentified but remained unredacted for study investigators.

The study was approved by the King Hussein Cancer Center’s institutional Review Board (IRB) and all enrolled patients signed a consent form. Eligibility for genetic testing as per the current guidelines was documented for each tested patient in order to assess and compare data of patients tested based on universal testing only vs. guideline-based eligibility testing. 

### Statistical Analysis

Means, median, standard deviations, and proportions were acquired using descriptive statistics. We included both pathogenic and likely pathogenic variants, reported as both numbers and percentages. The analysis also included patients with variants of uncertain significance (VUS). We examined the possible association between certain disease characteristics, like disease stage and specific pathological type, and patient demographics like age at diagnosis and family history with P/LP variant detection rate. The proportion of P/LP mutation in each comparison group was compared by Fisher’s exact test, with *p*-values < 0.05 indicating a statistically significant difference. All statistical analyses were performed using STATA version-18.

## 3. Results

Between March 2021 and December 2023, a total of 130 patients with a confirmed diagnosis of endometrial cancer were enrolled. A total of 64 (49.2%) patients were eligible for testing as per the NCCN guidelines, while 66 (50.8%) were not eligible and were tested as part of a universal germline genetic testing protocol which was running at the institution at the same time. The median age at diagnosis was 60 (range from 22–89) years; 27 (20.8%) patients were 50 years old or younger and, except for two, were all Jordanians. Endometrioid adenocarcinoma was the most common histological type (n = 74, 56.9%), while 20 (15.4%) patients had serous endometrial carcinoma and 10 (7.7%) patients had carcinosarcoma. Most of the patients enrolled had early-stage disease at diagnosis, with 37 (28.5%) patients presenting with metastatic disease. Lynch-related DNA mismatch repair alteration in tissue specimens was identified in a total of 61 (46.9%) patients, mostly in *PMS2* (n = 30, 23.1%) and *MLH1* (n = 22, 16.9%), Table 1.

**Figure 1:**
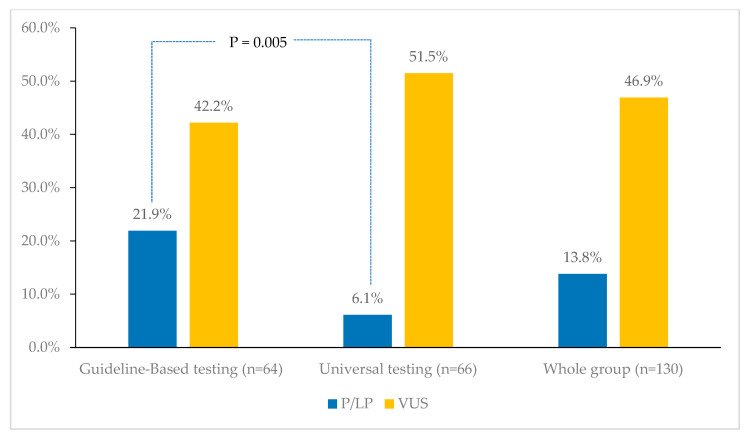
Frequency of P/LP and VUS among the study group. The rate of P/LP variants was significantly higher (21.9%) among patients who were eligible for testing (guideline-based) compared to those who were tested based on the universal testing protocol (6.1%). P/LP: pathogenic/likely pathogenic; VUS: variants of uncertain significance.

Among the whole group, 18 (13.8%) patients tested positive for pathogenic/likely pathogenic (P/LP) germline gene mutations. The rate of P/LP variants was significantly higher (n = 14, 21.9%) among patients who were eligible for testing (n = 64) compared to those (n = 66) who were tested based on the universal testing protocol (n = 4, 6.1%), *p* = 0.005 (Figure 1). 

**Figure 2:**
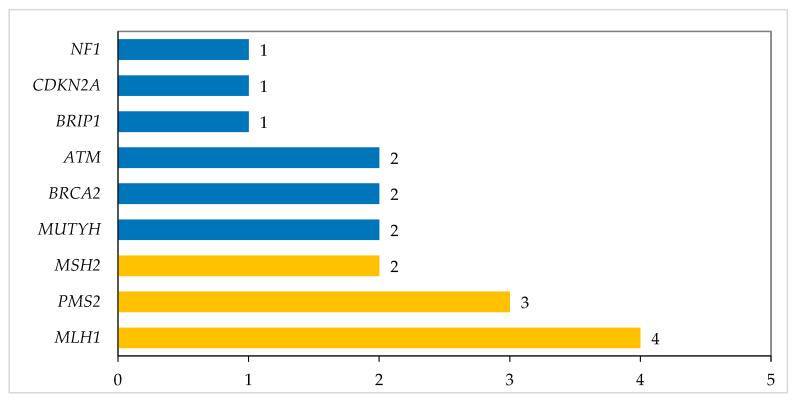
Pathogenic/likely pathogenic variants.

The mutations identified were: *MLH1* (four patients), *PMS2* (three patients), *MUTYH* (two patients), *MSH2* (two patients), *BRCA2* (two patients), *ATM* (two patients), *BRIP1*, *CDKN2A,* and *NF1* (one each). Additionally, seven (5.4%) other patients were found to be carriers of *MUTYH* (n = 3; 2.3%), *MSH3* (n = 2; 1.5%), *NTHL1*, and *FH* (one patient each); five of them were non-eligible for testing. In addition, three (2.3%) patients were found to have an increased risk allele of the *APC* gene, such patients being in the non-eligible group (Figure 2). 

**Figure 3:**
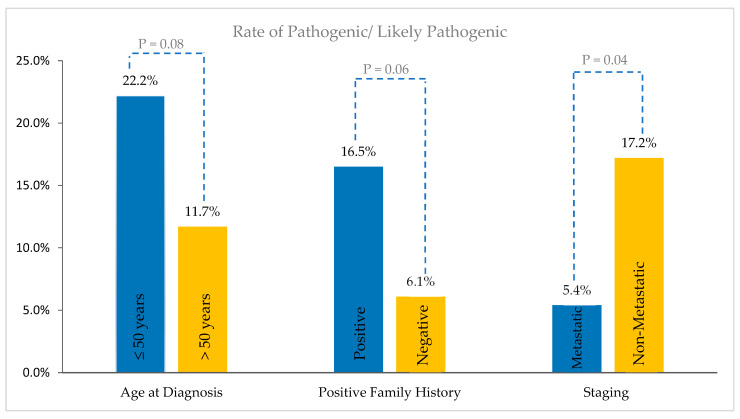
Frequency of pathogenic/likely pathogenic (P/LP) and variants of uncertain significance (VUS) study subgroups. P/LP variants were significantly higher among patients with early-stage disease and numerically higher in patients with a family history of cancer as well as among younger patients (age ≤ 50 years).

Pathogenic/likely pathogenic variants were significantly higher among patients with early-stage disease compared to those with metastatic disease, 17.2% versus 5.4%, *p* = 0.04, they were numerically higher in patients with a family history of cancer (16.5%) compared to patients without (6.1%), *p* = 0.06, and they were higher among younger patients (age ≤ 50 years), i.e., 22.2% compared to 11.7% among older ones, *p* = 0.08 (Figure 3). 

**Figure 4:**
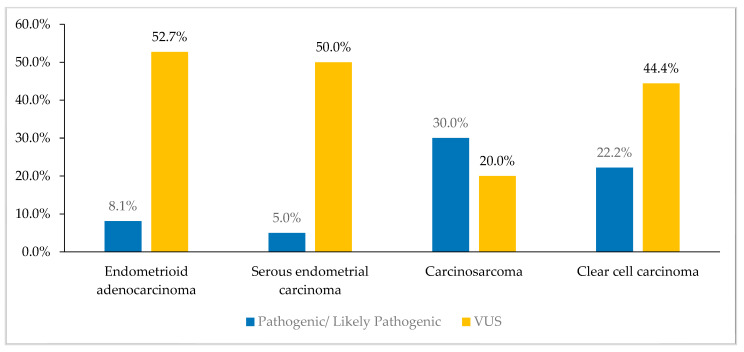
Pathogenic/likely pathogenic (P/LP) and variants of uncertain significance (VUS) based on pathological subtype. P/LP variants were more common among patients with carcinosarcoma (30%) and clear cell carcinoma (20.0%) compared to patients with endometroid adenocarcinoma (8.1%) or serous endometrial carcinoma (5%).

Additionally, P/LP variants were more common among patients with carcinosarcoma (30%) and clear cell carcinoma (22.2%) compared to patients with endometroid adenocarcinoma (8.1%) and serous endometrial carcinoma (5%), *p* = 0.8, Figure 4.

A total of 76 (58.5%) patients had identified variants of uncertain significance (VUS), many exhibiting more than one variant. The total number of VUS mutations identified among the study group was 118; 15 (12.7%) of these mutations were identified in patients who had another P/LP, increased risk allele, or carrier variants. Among the 61 (46.9%) patients who had only VUS gene mutations, the majority (n = 34, 55.7%) were identified based on the universal testing. The most commonly identified VUS mutations were *POLE*, *MSH3*, and *POLD1* (n = 5, 8.2% each). 

## 4. Discussion

Cancer-predisposing germline gene mutations, mostly in *BRCA1* and *BRCA2*, are better linked to breast and ovarian cancers [28]. Endometrial cancer, along with colon cancer, can be part of the well-described cancer-predisposing Lynch syndrome. In addition to its role in preventing the occurrence of various cancers, germline genetic testing for inherited cancer predisposition may inform patient care, including decisions on surgical options, radiation therapy, chemotherapy, immunotherapy, and targeted therapy [29,30]. In our study, we show that germline genetic variants, mostly in genes related to the Lynch syndrome, are relatively common among Arab patients with endometrial cancer.

Patients with a significant family history of endometrial and/or colorectal cancer (even with respect to patients without MMR defects who are MSI-stable or patients without screening) should be referred for genetic counseling and evaluation. Additionally, screening for genetic mutations should be considered, especially for patients younger than 50 years of age [18].

Depending on disease stage, patient’s age, and desire for fertility, surgical options for patients with newly diagnosed endometrial cancer may include a hysterectomy with a bilateral salpingo-oophorectomy, too. The likelihood of a heritable genetic variant at the time of endometrial cancer diagnosis may be quantified by utilizing the IHC of endometrial sampling. Recently, genetic testing has become an integral part of daily oncology practice with respect to many cancers, including endometrial. Appropriate pre- and post-testing genetic counselling are paramount to a successful outcome. The last decade has witnessed a revolution with respect to how we treat many cancers including those affecting the endometrium. Immune checkpoint inhibitors are now approved for use in endometrial cancers that have MSI or MMR deficiency [31].

In our study, we showed a relatively high frequency of germline variants, mostly in genes related to the Lynch syndrome. Similarly to studies on Western populations, mutations in genes other than those associated with the Lynch syndrome, including *MUTYH, BRCA2, ATM*, *BRIP1, CDKN2A,* and *NF1*, were identified [20,21,22,23,24]. The risk of endometrial cancer in women with P/LP *BRCA1* or *BRCA2* variants is not well characterized. Only two patients with *BRCA2* and none with *BRCA1* were identified in our cohort. Researchers have tried to draw an analogy between the endometrial carcinoma, mainly the serous subtype, and the serous ovarian carcinoma, the latter being well known for its association with a positive family history and germline mutations in *BRCA1/2*. Some studies have reported a positive family history in approximately 10% of cases of endometrial cancer, suggesting a possible inherited predisposition [32]. In a systematic review that intended to summarize the findings of all published studies on the prevalence of *BRCA1/2* mutation in patients with endometrial cancer, a total of 1613 patients enrolled into 11 clinical studies were tested for *BRCA1/2* mutations and were included in the analysis. *BRCA1/2* were identified in 70 (4.3%) women with endometrial cancers, while *BRCA1* was identified in 71.4% of the patients [33]. Likewise, the authors also reviewed the incidence of endometrial cancer in patients with *BRCA1/2* pathogenic variants, knowledge that should help patients and surgeons alike consider conducting a hysterectomy at the time of salpingo-oophorectomy for such patients. In this analysis, 209 patients with *BRCA1/2* variants reported in 14 studies were diagnosed with endometrial cancer. A correlation between *BRCA1/2* P/LP variants and endometrial cancer was reported in only five of the 14 studies; such a correlation was related to the *BRCA1* variant and not to *BRCA2* in two studies [16]. The advantages of performing a hysterectomy at the time of a risk-reducing salpingo-oophorectomy (RRSO) should also be taken into account if the patient is to receive hormonal therapy for the management of premature menopausal symptoms and for the minimization of cardiovascular disease and bone mineral loss following the bilateral salpingo-oophorectomy [34,35,36,37] or if the patient is to be treated with tamoxifen as an adjuvant therapy for early-stage breast cancer or as a form of chemoprevention [38] for individuals at high-risk of developing breast cancer, like those with *BRCA1/2* who opt to keep their breast.

Patients with confirmed P/LP alterations in one of the Lynch genes should be screened for associated cancers. Screening recommendations are clearer for colorectal cancer than for the gynecological cancers, as less information is available regarding tools and frequency of screening in such patients [39,40,41,42].

The relatives of patients with the Lynch syndrome should be tested for genetic mutations and, when positive, relatives may undergo a yearly endometrial biopsy to assess the risk of developing cancer [43]. Such a strategy enables select patients to defer surgery and preserve fertility until they complete their family, following which a prophylactic hysterectomy and BSO are highly recommended. 

Though our study represents the first attempt to study the landscape of germline mutations in patients with endometrial cancer in our region, the relatively small number of patients included and the fact that most of the patients were from one country and from a single center may limit our ability to generalize these findings to all “Arabs”. Larger multinational studies are needed to link treatment outcomes with specific germline alterations.

## 5. Conclusions

Similarly to Western societies, germline genetic variants, mostly in genes related to the Lynch Syndrome, are relatively common among Arab patients. The association of other pathogenic variants encountered with endometrial cancer is difficult to establish. Larger studies are needed to study the impact of various germline pathogenic variants on treatment outcomes.

## Figures and Tables

**Table 1:** Patients characteristics (n = 130).

Characteristics		Number	Percentage
Age at diagnosis (years)	Median (range)	60	
	≤ 50 years	27	20.8
	> 50 years	103	79.2
Positive family history		97	74.6
Nationality	Jordanians	128	98.5
	Non-Jordanians	2	1.5
Pathology	Endometrioid adenocarcinoma	74	56.9
	Serous endometrial carcinoma	20	15.4
	Carcinosarcoma	10	7.7
	Clear cell carcinoma	9	6.9
	Others	17	13.1
Metastases	Present	37	28.5
	Absent	93	71.5
MMR Status	PMS2	30	23.1
	MLH1	22	16.9
	MSH2	4	3.1
	MSH6	5	3.8

MMR: Mismatch-repair.

## Data Availability

Data used to generate this manuscript can be made available through the corresponding author upon reasonable request. All variants, including those reported in this manuscript, are submitted regularly to ClinVar. Details are available at: https://www.ncbi.nlm.nih.gov/clinvar/submitters/500031, (accessed on 1 March 2024).

## Supplementary Materials

The following supporting information can be downloaded at: https://www.mdpi.com/article/10.3390/cancers16142543/s1, Table S1: List of tested genes.
