# Supplementary material for: Patterns and Frequency of Pathogenic Germline Mutations among Patients with Newly-Diagnosed Endometrial Cancer: The Jordanian Exploratory Cancer Genetics (Jo-ECAG) Endometrial Study
Source: Cancers (Basel). 2024 Jul 15;16(14):2543. doi: 10.3390/cancers16142543 (PMC11274358; doi:10.3390/cancers16142543)
Supplement: Supplementary file 1 [file cancers-16-02543-s001.zip › cancers-3064604-supplementary.pdf]

Table S1. List of Tested Genes

Endometrial guided-gene panel test:

APC
AXIN2
BMPR1A
CHEK2
EPCAM
GREM1
MBD4
MLH1
MLH3
MSH2
MSH3
MSH6
MUTYH
NTHL1
PMS2
POLD1
POLE
PTEN
RNF43
SMAD4
STK11
TP53

Expanded multi-cancer panel test:

The full 84 multi-cancer genes panel:

AIP
ALK
APC
ATM
AXIN2
BAP1
BARD1
BLM
BMPR1A
BRCA1
BRCA2
BRIP1
CASR
CDC73
CDH1
CDK4
CDKN1B
CDKN1C
CDKN2A

CEBPA
CHEK2
CTNNA1
DICER1
DIS3L2
EGFR
EPCAM
FH
FLCN
GATA2
GPC3
GREM1
HOXB13
HRAS
KIT
MAX
MEN1
MET
MITF
MLH1
MSH2
MSH3
MSH6
MUTYH
NBN
NF1
NF2
NTHL1
PALB2
PDGFRA
PHOX2B
PMS2
POLD1
POLE
POT1
PRKAR1A
PTCH1
PTEN
RAD50
RAD51C
RAD51D
RB1
RECQL4
RET
RUNX1
SDHA
SDHAF2
SDHB
SDHC
SDHD

SMAD4
SMARCA4
SMARCB1
SMARCE1
STK11
SUFU
TERC
TERT
TMEM127
TP53
TSC1
TSC2
VHL
WRN
WT1
